# Influences of metabolism and lipid homeostasis on regulatory vs. conventional T cells and implications for autoimmunity

**DOI:** 10.3389/fimmu.2025.1613230

**Published:** 2025-07-07

**Authors:** Madison A. Nguyen, Sarah S. Lee, Craig M. Walsh

**Affiliations:** ^1^ Molecular Biology and Biochemistry Department, Charlie Dunlop School of Biological Sciences, University of California, Irvine, Irvine, CA, United States; ^2^ Sue & Bill Gross Stem Cell Research Center, University of California, Irvine, Irvine, CA, United States; ^3^ Institute for Immunology, University of California, Irvine, Irvine, CA, United States

**Keywords:** regulatory T cells, lipid metabolism, metabolic reprograming, mevalonate (MVA) pathway, mTOR, autoimmune disease

## Abstract

Regulatory T cells are essential for suppressing an overactive immune system, especially concerning autoimmune disease, tumor growth, and inflammatory disease. This suppressive nature of regulatory T cells is largely due to their metabolic profiles determined by metabolic reprogramming upon activation and subsequent differentiation. As regulatory T cells tend to process and cycle energy differently from other T cell subsets, we are interested in what metabolic processes support regulatory T cell function. This review will consider how regulatory T cells compare with conventional T cells in terms of their participation in distinct metabolic pathways and how the presence of regulatory T cell-specific molecules influences proliferation and suppressive function. Additionally, this review will identify possible metabolic targets of regulatory T cells that could be targeted for development of autoimmune disease therapies.

## 1 Introduction

T lymphocytes are an integral part of the human adaptive immune system and play an essential role in the control of autoimmune disease. Upon activation, T cells undergo metabolic reprogramming, resulting in a shift in reliance on different metabolic pathways that can determine T cell lineage and function due to changes in how energy is generated and processed. These metabolic pathways can be classified as either catabolic, involving the breakdown of molecules, or anabolic, the construction of molecules. For example, the synthesis of nucleic acids and proteins is an anabolic pathway and is important for activated T cells that have an anabolic metabolism due to promoting enhanced cell division that requires metabolic output and reprogramming after activation (1). At an evolutionary level, the adaptive immune system must respond to invasion by foreign pathogens after initial action by the innate immune system, a process necessitating metabolic reprogramming of T cells. Engagement of different metabolic pathways in T cells allows them to meet energetic needs to grow, proliferate, and acquire effectors functions as critical components to fighting an infection (2). Similar metabolic reprogramming is necessary for anti-tumor immunity.

Lipid metabolic processes are especially vital for the breakdown and usage of fatty acids to produce energy for T cells which most prominently participate in processes such as aerobic glycolysis, fatty acid β-oxidation (FAO), oxidative phosphorylation (OXPHOS), and *de novo* lipogenesis (DNL) (2–5). For example, while T cells use both OXPHOS and aerobic glycolysis, only OXPHOS is necessary for naïve T cell activation, while a shift to aerobic glycolysis supports effector function (6). Such processes are essential to T cell survival and function and can influence fate for differentiation of CD4 or CD8 T cell subsets including naïve, effector, memory, or regulatory T cells (Tregs).

Tregs expressing CD4, CD25, and forkhead box P3 (FOXP3) help regulate immune response and homeostasis of the immune system and upon activation, experience distinct metabolic reprogramming from conventional T cells, enabling their unique and essential function. This review discusses how Tregs differ from conventional T cells when considering their metabolic profiles and analyzes how these differences can be used in application towards therapeutics and progress in autoimmune disease.

## 2 Metabolic profiles of conventional T Cells

### 2.1 Metabolic reprogramming in CD4 v. CD8 T cells support effector function and growth after activation

**Figure 1:**
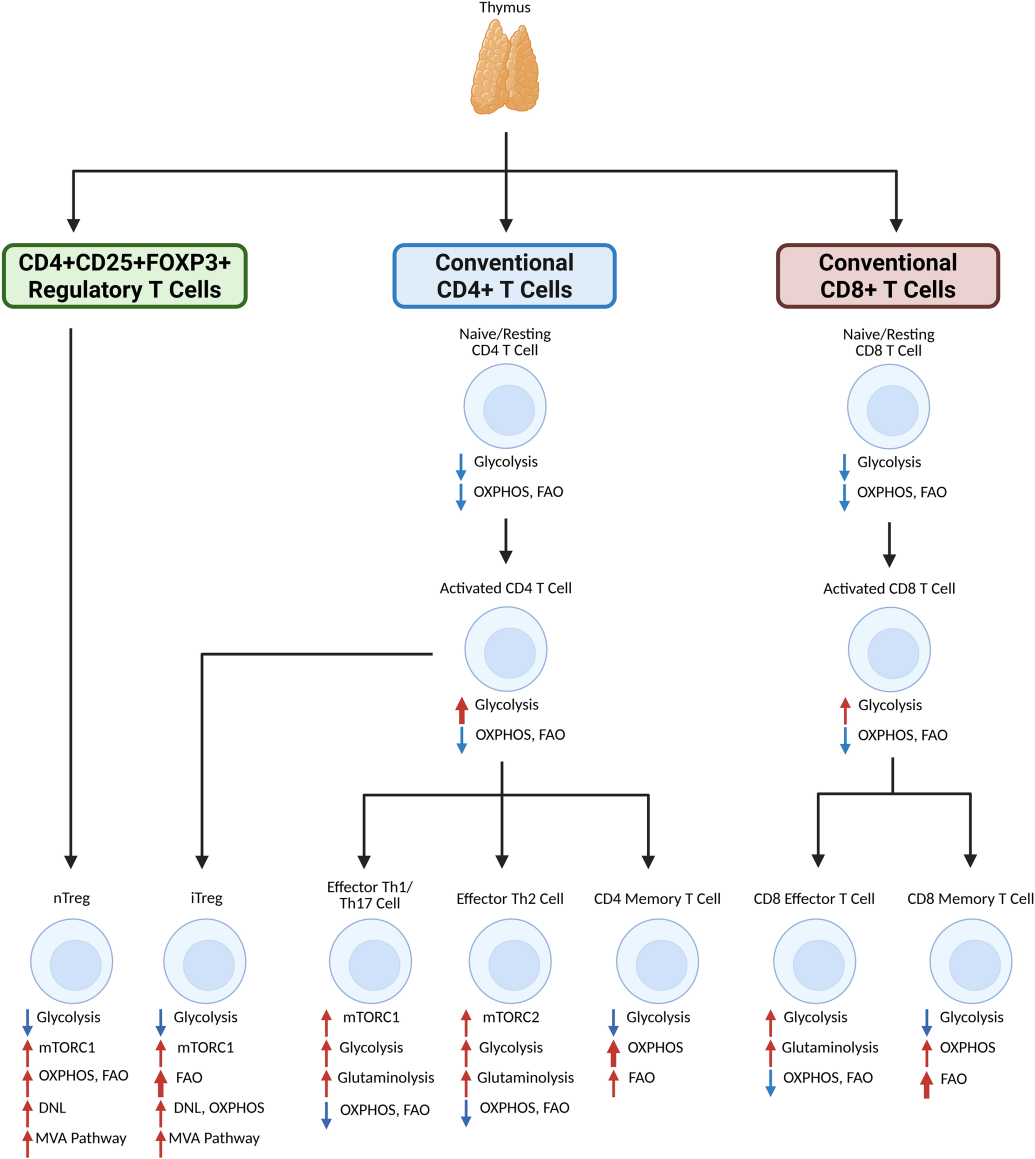
Generalized overview of primary lipid metabolism participation and metabolic reprogramming of T cell subsets upon activation and differentiation. Inactivated CD4 or CD8 T cells that originate from the thymus do not rely heavily on metabolism for function. Upon activation, a shift in glycolysis is observed, especially for CD4 T cells, while OXPHOS and FAO levels remain stable. CD4 memory T cells lessen reliance on glycolysis in favor of FAO and especially OXPHOS, whereas CD8 memory T cells similarly shift reliance to OXPHOS and especially FAO. CD8 effector T cells have enhanced glycolysis and glutaminolysis. CD4 effector Th1 and Th17 cells rely on glycolysis and glutaminolysis after differentiation via mTORC1, and CD4 effector Th2 cells also rely on glycolysis and glutaminolysis after differentiation, but via mTORC2. Regulatory T cells, which reprogram via mTORC1 and the MVA pathways to rely more on OXPHOS, FAO, and DNL. iTregs, which differentiate from activated CD4 T cells, rely on FAO more than nTregs, which differentiate from the thymus. Created in BioRender. Nguyen, M. (2025) https://BioRender.com/la11dgt.

Naïve T cells have a catabolic metabolism while activated T cells shift to anabolic metabolism through the Warburg effect which describes a shift from OXPHOS to aerobic glycolysis (**Figure 1**). In correlation to T cells, the Warburg effect occurs in response to T cell activation from CD28 costimulatory signaling, which increases glucose uptake via upregulation of aerobic glycolysis through phosphatidylinositol 3‐kinase (PI3K) mediated glucose transport expression (7). In activated T cells, fatty acid production via DNL and isoprenoid and cholesterol production via sterol regulatory element-binding protein (SREBP) transcription factors in the mevalonate pathway (MVA) is also upregulated to generate energy resources needed for clonal expansion and activation following T cell receptor (TCR) engagement (5, 8).

Naïve CD4 and CD8 T cells primarily rely on low levels of OXPHOS and FAO to remain in a quiescent state before activation and Myc-driven reprogramming (9). Subsequently, the presence of key molecules involved in metabolic processes is vital to fate determination for CD4 T cell subsets. From a study by Berod et al., inhibition of acetyl coenzyme A carboxylase 1 (ACC-1) mediated DNL favors Treg formation over T helper 17 (Th17) T cell formation (10). Upon activation, CD8 T cells rely on glycolysis to a lesser degree than CD4 T cells (11). This differentiation is also impacted by mechanistic target of rapamycin complexes (mTORC1 and mTORC2). mTORC1 and mTORC2 pathways act as metabolic sensors, which can promote differentiation of CD4 T cells into Th1, Th17 (via mTORC1), and Th2 (via mTORC2) effector cells while shifting reliance towards glycolysis and glutaminolysis (12–14). These pathways additionally negatively regulate memory CD8 T cell differentiation in favor of effector CD8 T cells (15).

Other molecules involved in metabolic processes are integral to T cell survival and functions. One protein, CD36, is a scavenger receptor involved in the uptake of fatty acids and mediates lipid peroxidation in T cells (16). Ferroptosis mediated by CD36 also serves to suppress CD4 and CD8 T cell effector function (17, 18). Additionally, fatty acid binding proteins (FABPs) are involved in general T cell homeostasis and metabolism. T cells primarily express FABP5/E-FABP, which can influence T cell function and survival through upregulation of fatty acid uptake, particularly linoleic acid (19, 20). TCR signaling is also affected by lipid metabolism through lipids binding to transcription factors, such as fatty acids activating peroxisome proliferator-activated receptors (PPARs) PPAR-α, PPAR-β and PPAR-γ, which in turn influence T cell responses (21–23). Lipid metabolism is vital for maintaining T cell function through metabolic reprogramming and is critical during infections or cases of autoimmunity by regulating differentiation. With Tregs, these levels of proteins and small molecules are regulated differently than in conventional T cells and impact their suppressive function, as discussed below.

### 2.2 Differences between naïve, memory, and effector T cell lipid metabolism

Subsets of CD4 and CD8 T cells require different levels of metabolic activity for different processes to function. Following thymic selection, conventional T cells can differentiate into naïve T cells, which are resting T cells; memory T cells, which are long-lived T cells able to recognize previously encountered antigens; or effector T cells, which are active in fighting off microbial pathogens and tumor cells. Naïve T cells have a low level of metabolic activity, simply maintaining basal levels of OXPHOS and FAO for survival while awaiting encounter with antigens before effector differentiation (9). Memory T cells maintain reliance on OXPHOS and FAO but upregulate metabolic activity over time to maintain a consistent energy supply, thereby supporting their long-term survival and possible re-exposure to antigens (24–26). Effector T cells, however, do shift from OXPHOS and FAO to glycolysis and glutaminolysis through key regulators such as those associated with the AMP-activated protein kinase (AMPK) pathway (27–30). This shift is responsible for maintaining proper effector function as well as for supporting continuous and rapid clonal expansion of T cells during infection and especially in cancer (6, 31).

## 3 Metabolic profile of regulatory T cells

### 3.1 Metabolic reprogramming in regulatory T cells supports suppressive function and homeostasis

**Figure 2:**
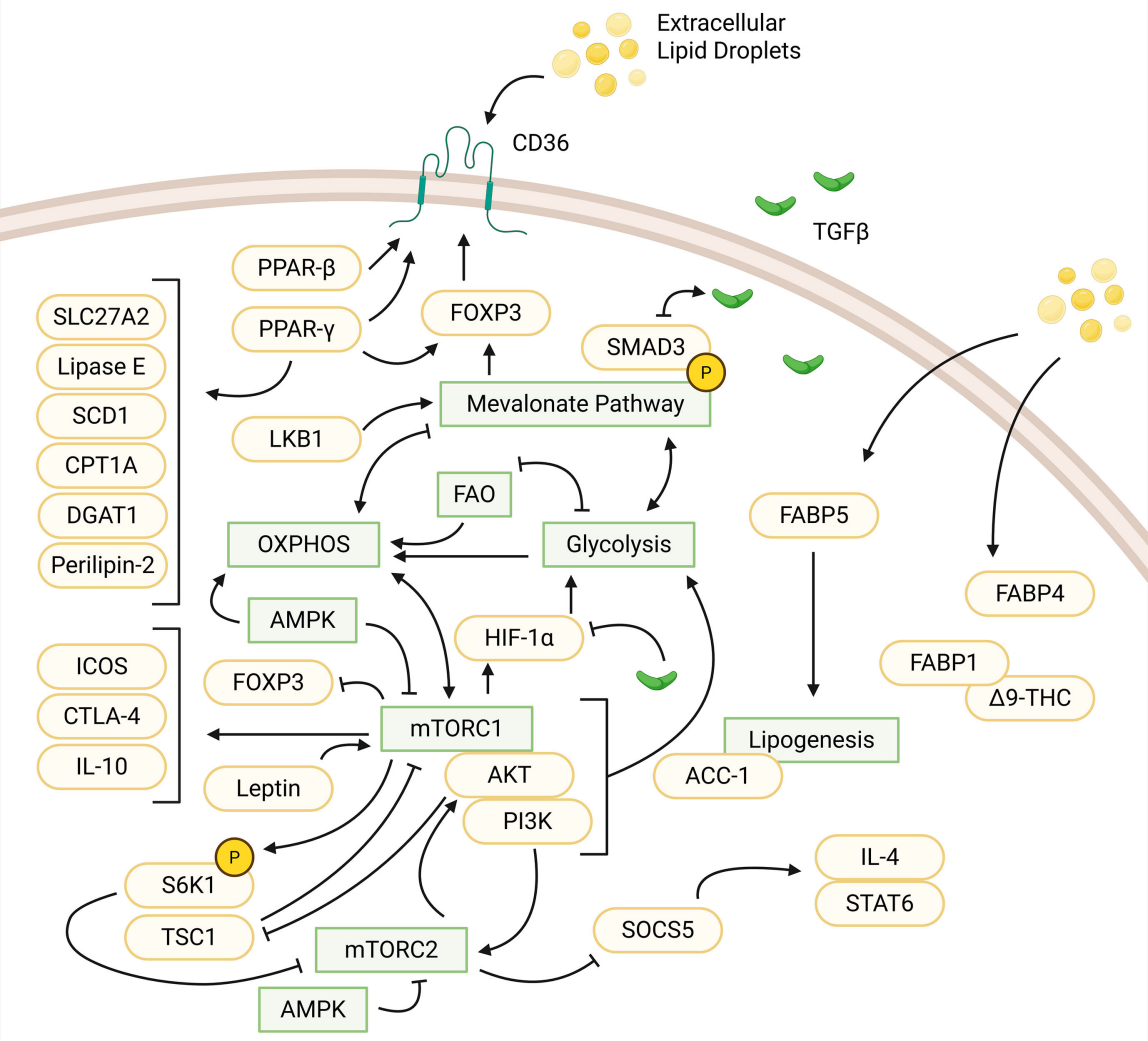
Influences of protein, small molecules, and metabolic pathways in regulatory T cells. Extracellular lipid droplets are uptaken, facilitated by CD36. CD36 activity is upregulated by PPAR-β and PPAR-γ (via interactions with FOXP3). PPAR-γ upregulates other small molecules: SLC27A2, lipase E, SCD1, CPT1A, DGAT1, and perilipin-2. LKB1 regulates the mevalonate pathway, which upregulates FOXP3 expression and phosphorylates SMAD3 to enhance TGFβ signaling. TGFβ signaling, in turn, downregulates SMAD3 expression. The MVA pathway is connected to various pathways indirectly. This includes a positive feedback loop between glycolysis and the MVA pathway. The MVA pathway activates OXPHOS while OXPHOS inhibits the MVA pathway. FAO and glycolysis have a negative feedback loop and both feed into OXPHOS. mTORC1 suppresses FOXP3 expression. OXPHOS and mTORC1 have a positive feedback loop while mTORC1 produces HIF-1α activating glycolysis. TGFβ is also a suppressor of HIF-1α. mTORC1 upregulates IL-10, ICOS, and CTLA-4. Leptin can form a complex with mTORC1 to activate the pathway. mTORC1 can form another complex, PI3K-AKT-mTORC1 to activate glycolysis. In relation to mTORC2, PI3K activates AKT, which suppresses TSC1 to inhibit mTORC1. mTORC1 phosphorylates S6K1, which inhibits mTORC2. mTORC2 suppresses SOCS5 to activate IL-4/STAT6 signaling. AMPK inhibits mTORC1 and mTORC2 to upregulate OXPHOS. FABP5 and FABP4 also facilitate uptake of extracellular lipids. FABP5 feeds into lipogenesis mediated by ACC-1. FABP1-Δ9-THC forms a complex within regulatory T cells. Created in BioRender. Nguyen, M. (2025) https://BioRender.com/me864o9.

Tregs are a subset of CD4 T cells whose suppression of autoreactive T cells impacts autoimmune and inflammatory diseases. Tregs express FOXP3 and key inhibitory cytokines such as transforming growth factor-β (TGF-β), interleukin-10 (IL-10), and IL-35 (32–34). In particular, TGF-β1 is important for lipid metabolism and Treg generation due to TGF-β1 signaling upregulating mitochondrial fusion via the suppressor of mothers against decapentaplegic 2/3/peroxisome proliferator-activated receptor γ coactivator 1-α (SMAD2/3)/(PGC-1α) pathways and switching T cells from glycolysis to FAO by inhibiting hypoxia-inducible factor 1-α (HIF-1α) expression (35). This metabolic shift is reminiscent of the metabolism of memory T cells as Tregs also upregulate OXPHOS and FAO upon differentiation (**Figure 2**). Tregs are often found in low-glucose and high-lactate environments and use FOXP3 to initiate this reprogramming to sustain their suppressive function in such environments (36). This shift also differs, albeit to a lesser degree, between the metabolic profiles of induced Tregs (iTregs) and thymically derived natural Tregs (nTregs) particularly during proliferative states, where iTregs tend to be reliant on both glycolysis and FAO while nTregs are primarily glycolytic (37).

Through metabolic sensors regulated by mTORC1 and mTORC2 signaling, Tregs can also utilize both intracellular and extracellular lipids for different mechanisms maintaining homeostasis (38). For instance, Tregs have enhanced DNL to generate intracellular fatty acids, enhanced breakdown of mevalonate into ceramides, isoprenoids, and cholesterols, and increased extracellular lipid uptake via CD36 and FABP5 (39–42). Fatty acids are also important for Tregs in the colon and promote the stability of FOXP3 protein when compared to general splenic Tregs (43).

### 3.2 The mevalonate pathway is essential for regulatory T cell activation and function

Mevalonate is an intermediate molecule in the MVA pathway responsible for signaling, T cell fate and lipid transportation through production of isoprenoids and cholesterols (8, 44). This pathway is essential for generating Tregs without affecting Th1 and Th17 differentiation through increased TGF-β signaling via enhanced SMAD3 phosphorylation that then promotes FOXP3 expression (40, 43, 45). In experiments by Acharya et al., mevalonate-treated Tregs appeared to have greater suppressive function against effector T cells than untreated cells, indicating that mevalonate is a positive regulator of Treg differentiation and suppressive function (40). Another study by Timilshina et al. highlights the importance of liver kinase B1 (LKB1) as a key regulator of the MVA pathway. LKB1 was shown to upregulate mevalonate gene expression, with the absence of LKB1 in Tregs causing autoinflammation through downregulation of important cytokines such as IL-10 without affecting the production of Th1 and Th17 cytokines (46). The MVA pathway is thus crucial to Treg activation without compromising conventional T cell activation.

### 3.3 Differences in mTORC1 and mTORC2 pathways in regulatory T cells

The mTOR pathway is a key regulator for cellular processes and consists of two multiprotein complexes, mTORC1 and mTORC2. mTORC1 regulates cell growth and metabolism through anabolic processes such as protein synthesis, and mTORC2 regulates cell survival and proliferation via catabolic processes and pathways such as PI3K-protein kinase B (AKT) phosphorylation and growth factor signaling (47, 48). For Tregs, reliance on the mTOR pathways is rather complex and understudied, as they can act as both a suppressor and activator of Treg function and a suppressor of Treg generation. While both mTORC1 and mTORC2 are important for Treg and conventional T cell differentiation, only mTORC1 is necessary for Treg suppressive function upon differentiation (49). This is due to interactions of the PI3K-AKT-mTORC1 signaling pathway, which primarily activates glycolysis in T cells and impairs Treg proliferation (50, 51). Upregulation of mTORC1 signaling also influences Treg suppressive function by supporting the mevalonate pathway through HIF-1α, which promotes enhanced lipid metabolism and maintenance of suppressive factors such as IL-10, inducible T cell costimulator (ICOS), and cytotoxic T-lymphocyte associated protein 4 (CTLA-4) (52–55). However, mTORC1 can also negatively regulate FOXP3 expression in T cells as seen in mice with both a Rheb deficiency and lack of mTORC1 activation, leading to a loss of suppressive function (56). mTORC1 is a key regulator of suppressive function, but overactivation of mTORC1 can be detrimental. In naïve T cells, overactivation can lead to impairments in Treg differentiation, as mTORC1 upregulates glycolytic processes that favor conventional effector T cells, particularly Th1 and Th17 cells (57). This overactivation can be controlled by factors such as AMPK activation, which inhibits mTORC1 to promote OXPHOS and FAO through regulation of metabolite transcriptional control factors such as PGC-1a and HIF-1a (58). Additionally, introducing rapamycin to T cells, which inhibits both mTORC1 and mTORC2 (although more weakly for the latter), is shown to directly affect T cell differentiation and selectively expand Tregs *in vitro* (59).

In Tregs, mTORC2 is primarily associated with homeostatic regulation and Treg differentiation but it can also indirectly maintain suppressive function by stabilizing FOXP3 (52). Regulation of the Treg and Th2 cell balance is notably maintained through an mTORC2 signaling complex that inhibits suppressor of cytokine signaling 5 (SOCS5), which supports IL-4/signal transducer and activator of transcription 6 (STAT6) signaling to favor differentiation of Th2 cells (60). To combat overactivation, the AMPK pathway not only contributes to suppression of mTORC1 but also suppression of mTORC2 to maintain Treg balance and catabolism (61). mTORC2 activity also regulates Treg differentiation in the thymus in the absence of tuberous sclerosis complex-1 (TSC1), which negatively regulates mTORC1, and deletion results in overactivation of mTORC1 (62). This overactivity of mTORC1 again leads to a loss in suppressive activity to favor effector-like function, with TSC1-deficient cells losing FOXP3 expression and producing proinflammatory cytokines such as IL-4 or IL-17 due to decreased levels of ribosomal protein S6 kinase 1 (S6K1) production (55, 62, 63). While both mTORC1 and mTORC2 complexes contribute negatively to the generation of Tregs in favor of effector T cells, they also play intricate roles in supporting and impairing the suppressive function of differentiated Tregs.

### 3.4 Contributions of enzymes, receptors, and proteins involved in regulatory T cell lipid metabolism

Besides signaling pathways, the metabolic profile of Tregs is influenced by the upregulation and downregulation of certain proteins and differ between tissue types. CD36 is one such protein that promotes Treg proliferation at the expense of CD8 T cell function through a PPAR-β dependent mechanism increasing lipid peroxidation in CD8 T cells, particularly in the tumor microenvironment (TME) (16, 41). Another PPAR, PPAR-γ is a receptor found in Tregs involved in the upregulation of FAO through CD36/carnitine palmitoyltransferase 1 (CPT1)-activated enzymes (64). This function occurs to a larger extent in visceral adipose tissue (VAT-tissue) than splenic tissue, where PPAR-γ interacts with FOXP3 to upregulate fatty acid transporters such as CD36 or solute carrier family 27 member 2 (SLC27A2), enzymes involved in fatty acid synthesis such as lipase E and stearoyl-CoA desaturase 1 (SCD1), an enzyme essential for fatty acid oxidation. Additional enzymes in this pathway include CPT1a, a CPT1 isoform enzyme involved in the synthesis of triglycerides, diacylglycerol-acyltransferase 1 (DGAT1), and a protein perilipin-2 that is associated with lipid droplets (65). PPAR-γ in Tregs is responsible for enhanced lipid uptake in VAT-tissue via increased expression of CD36 levels, which can be important for disorders like type 2 diabetes and obesity (65–67).

FABPs are also involved in T cell metabolic regulation, including in Tregs due to FABP5-dependent uptake of extracellular fatty acids and increased lipogenesis (32). For Tregs, FABP5 levels tend to be greater when compared to naïve T cells. Previous studies examined Tregs after inhibition of FABP5 using BMS309403 which led to decreased OXPHOS, enhanced glycolysis, and altered mitochondrial cristae without compromising suppressive function (42). Alternatively, this inhibition appeared to enhance suppressive function through decreased lipid availability, with an increase in type 1 (interferon) IFN and IL-10 signaling (42). In the absence of FABP5 in Tregs, suppression function is enhanced and leads to the reduction of Th17 cell differentiation, thereby preventing development of autoimmune diseases such as experimental autoimmune encephalomyelitis (EAE) resistance, a mouse model of multiple sclerosis (68). Other FABPs are regulators of Treg differentiation and metabolism. For example, FABP4 is involved in lipid transport and influences the Treg/Th17 balance, whereas FABP1 regulates hepatic transport of Δ9-THC to promote Treg differentiation (69, 70). These proteins and other small molecules involved preferentially in Tregs are some of the key factors that dictate how they maintain Treg homeostasis and suppressive ability when coupled with metabolic reprogramming.

## 4 Why different T cell subsets might rely on distinct metabolic pathways

While it is relatively unknown why different T cells rely on distinct metabolic pathways, it can be inferred that this may be the result of differences in the suppressive response, pro-inflammatory effector functions and other key factors associated with T cell subset differentiation and function. Nutrient availability affects the means through which T cells process energy, and metabolites such as acetyl-CoA and lactate further shape the T cell environment and Treg balance (10, 71). These features indicate that T cells must be metabolically flexible to carry out their distinct functions in non-lymphoid tissues. Unlike conventional T cells that recognize antigen and rapidly proliferate during clonal expansion, Tregs do not necessarily require this function. We surmise that conventional effector T cells require a cellular metabolism that favors rapid clonal expansion (e.g. glycolysis) in order to generate sufficient microbial antigen specific clones to control an infection. Tregs would not need this rewiring as they mainly function as suppressors of T cell autoreactivity through cytokine production and thus would not need to consume an exorbitant amount of energy for clonal expansion unlike conventional T cells. The reliance of Tregs on metabolic pathways such as OXPHOS and FAO instead of glycolysis comes from factors such as phosphatase and tensin homolog deleted on chromosome 10 (PTEN) and FOXP3 expression initiating metabolic reprogramming, which may provide Tregs more metabolic control in glucose-depleted environments (36, 72). In general, metabolic reprogramming of T cells is speculated to be based on T cell functionality during antigen response as well as the surrounding nutrient availability within the target tissue.

## 5 Targeting regulatory T cell metabolism as a potential strategy for autoimmune disease therapies

The metabolic reprogramming of Tregs involves the switch in reliance of different metabolic pathways, and these differences might be harnessed for the development of autoimmune disease therapeutics. This concept of targeting distinct metabolic pathways has been applied in other areas such as cancer treatments. Tregs often contribute to tumor growth as they suppress tumor antigen specific conventional T cells, however, pharmacologic inhibition of factors that influence Treg function within the tumor could yield enhanced anti-tumor immunity. Tregs in the tumor microenvironment regulate metabolism by manipulating CD36-mediated uptake of free fatty acids and mTOR-mediated activity of amino acids pools such as serine via glutathione regulation (41, 73). Similarly, in autoimmune disease, Tregs have an altered metabolic pathway resulting in impaired mitochondrial function with reduced suppressive activity (74). In multiple sclerosis, leptin is upregulated and is associated with a reduction in Tregs (75, 76). This phenomenon alters mTOR pathway activation via leptin, which induces downregulation of IL-2 and thus a loss of Treg suppressive function (20, 52). With autoimmune diseases caused by autoreactive T cells that can be directly suppressed by Tregs, Treg generation and maintenance of suppressive function are key factors in controlling disease progression. By harnessing metabolic reprogramming to manipulate Treg metabolism, e.g. through the mevalonate or mTOR pathways, it may be possible to enhance Treg suppressive differentiation and/or function. Since such metabolic pathways are highly conserved in a variety of different tissues and cell types, the challenge will be to selectively target regulatory pathways that are distinct in Tregs and other T cell subsets to avoid adverse events.

## 6 Conclusions

Tregs are vital components of adaptive immunity, especially against autoimmune disease through the suppression of autoreactive T cells. Treg function differs from that of conventional T cells partly due to their distinct metabolic requirements, and these metabolic pathways alter the way Tregs generate and consume energy and influence their suppressive nature. With further understanding of the roles that distinct metabolic pathways play in Treg differentiation and function, further understanding of the specific proteins and signaling pathways that regulate cellular metabolism in Tregs and other T cell subsets may yield key insight for development of therapies to target autoimmunity, cancer and other diseases impacted by the adaptive immune system.
